# EHMTI-0298. Relationship between migraine and abnormal EEG findings in children

**DOI:** 10.1186/1129-2377-15-S1-B16

**Published:** 2014-09-18

**Authors:** P Karimzadeh

**Affiliations:** 1 Pediatric Neurology, shahid Beheshti Medical University, Tehran, Iran

## Introduction

Migraine is a disabling illness that causes absence from school and affects the quality of life. It has been stated that headache may represent an epileptic event. EEG abnormality is a prominent finding in children with migraine.

## Aim

The aim of this study was to evaluate EEG abnormalities in children with migraine.

## Materials and methods

Two-hundred twenty-eight children were enrolled into the study. Evaluation and following of cases was performed by one physician, paraclinical tests were used to increase the accuracy. The study was conducted under the supervision of pediatric neurology masters and the selected cases were from different parts of the country.

## Results

Comparing EEG abnormalities in different types of migraine revealed that there is an association between them. There was also a significant difference between EEG abnormalities in different types of aura. Migraine type was associated with the patient’s age. Sleep disorders were more common in patients with a positive family history of seizure.

## Conclusions

Our study disclosed migraine as a common problem in children with abnormalities present in approximately 20% of the patients. Migraine and abnormal EEG findings are significantly associated.

No conflict of interest.

